# Sleepiness but neither fluid nor crystallized intelligence can be predicted from resting-state electroencephalography – Evidence from the large scale CoScience EEG-Personality Project

**DOI:** 10.3758/s13415-025-01323-y

**Published:** 2025-07-01

**Authors:** Christoph Fruehlinger, Katharina Paul, Corinna Kührt, Jan Wacker

**Affiliations:** 1 Department of Differential Psychology and Psychological Assessment, Institute of Psychology, University of Hamburg, Von-Melle-Park-5, 20146 Hamburg, Germany; 2 Faculty of Psychology, Technische Universität Dresden, Zellescher Weg 17, 01069 Dresden, Germany

**Keywords:** Personality neuroscience, Intelligence, Sleepiness, MVPA, Resting-state electroencephalography

## Abstract

Previous electroencephalogram (EEG) studies linked measures of spectral power under rest and fluid intelligence; however, subsequent high-powered studies challenged this relationship. The present study aimed to address previous limitations (low statistical power, lack of preregistration) and investigated the predictability of intelligence measures from resting-state EEG in the CoScience data set (*N* = 772). Support vector regressions were applied to analyze 8 min of resting-state EEG with eyes open and closed before and after unrelated tasks. The decoding performance between the spectral power of 59 EEG channels within 30 frequency bins and fluid and crystallized intelligence, was evaluated with a tenfold cross-validation. We could not identify any meaningful associations between resting-state EEG spectral power and either fluid or crystallized intelligence—a null finding that is unlikely to be entirely due to a relatively modest restriction of fluid intelligence variance in our student sample. Moreover, we did replicate the previously reported association between state sleepiness and theta power, attesting to the integrity of the CoScience data set. Furthermore, the decomposition of the EEG signal into its periodic and aperiodic components revealed that the aperiodic offset parameter is significantly correlated with state sleepiness, emphasizing the relevance of aperiodic signal components in understanding states of alertness versus sleepiness.

**Supplementary Information:**

The online version contains supplementary material available at 10.3758/s13415-025-01323-y.

Intelligence is the general ability to learn, store knowledge, and adapt to novel environments (Sternberg, 2012). It plays a fundamental role in determining life outcomes, such as academic or occupational success and health (Wraw et al., 2015; Zisman & Ganzach, 2022). Fluid intelligence involves the ability to resolve problems through reasoning and different mental operations. Conversely, crystallized intelligence reflects the depth of the acquired knowledge through cultural and educational experiences (Cattell, 1963; Horn, 1968; McGrew, 2009). Building on this foundational understanding, researchers have long sought to identify the biological correlates of intelligence, focusing on the brain as a central determinant.

Studies utilizing neuroscientific methods, such as magnetic resonance imaging (MRI) or electroencephalography (EEG), have advanced this inquiry, linking measures of brain structure and function to cognitive abilities. For example, meta-analyses have identified a robust and potentially causal relationship between brain volume and intelligence (Gignac & Bates, 2017; Lee et al., 2019; Pietschnig et al., 2015), and a number of studies found positive associations between intelligence and activity in frontoparietal circuits as proposed by parieto-frontal integration theory (P-FIT; Basten et al., 2015; Jung & Haier, 2007).

While these findings are considered relatively robust, various additional associations reported previously (Doppelmayr et al., 2002; Jaušovec & Jaušovec, 2000; Stankova & Myshkin, 2016) are probably best considered somewhat more preliminary given pervasive replication problems in many areas of Psychology and Neuroscience (Open Science Collaboration, 2015; Wacker & Paul, 2022). For instance, several studies attempted to link cognitive abilities to measures of resting-state EEG (often implicitly) assuming that measuring brain activity in a relatively unconstrained state at rest avoids task-specific confounds and allows for the exploration of intrinsic neural dynamics that may underlie cognitive processes. Previous resting-state EEG studies attempted to relate spectral power and cognitive ability measures in an a-theoretical and data-driven way. Some of this work reported a positive association between resting-state alpha power (i.e., 8–13 Hz) and cognitive abilities or test performance (Doppelmayr et al., 2002; Grandy et al., 2013; Hanslmayr et al., 2005; Jaušovec & Jaušovec, 2000; Klimesch, 1999; Makowski & Troche, 2024; Stankova & Myshkin, 2016; Zoefel et al., 2011). Interestingly, some of these studies also found moderating effects of gender on the strength of this association (Doppelmayr et al., 2002; Jaušovec & Jaušovec, 2005, 2010). In comparison, other studies found an association between resting-state theta power (i.e., 4−8 Hz) and cognitive abilities, although the direction of this association is rather inconsistent (negative: Klimesch, 1999; Stankova & Myshkin, 2016; positive: Jaušovec & Jaušovec, 2000; Thatcher et al., 2005). Critically, studies in larger samples could not replicate the relationship between cognitive abilities or fluid intelligence and neither alpha power (*N* = 153, Ociepka et al., 2022), nor any other frequency band (Akdeniz, 2018). Analogous associations for crystallized intelligence have not been documented in the literature at all.

Given this highly inconsistent pattern of results, two major culprits of the replicability crisis are likely at play in this line of research area as well: Low statistical power and the flexibility of data collection, analysis, and reporting due to the lack of preregistrations combine to significantly increase the likelihood of false-positive results (Ioannidis, 2005; Szucs & Ioannidis, 2017). Studies with sufficiently large samples and detailed preregistrations are thus necessary to achieve more clarity.

Furthermore, a potentially important confound that may partly underlie the observed inconsistencies has not been systematically considered so far: Sleepiness has been shown to negatively affect fluid intelligence test performance while leaving crystallized intelligence measures unaffected (Alhola & Polo-Kantola, 2007; Quigley et al., 2000). Moreover, a meta-analysis confirmed that sleepiness was positively associated with increased activity between 4 and 13 Hz (i.e., theta and alpha power, Tran et al., 2020). Thus, controlling for state sleepiness seems advisable when aiming to probe associations between EEG spectral power and intelligence.

Finally, to better understand the complex associations between EEG spectral power and cognitive abilities, it is essential to employ advanced analytical techniques capable of revealing subtle and multivariate patterns. A powerful method to analyze such relationships is multivariate pattern analysis (MVPA). It estimates the decoding or prediction performance between a multivariate input (e.g., EEG power from multiple channels) and an output variable (e.g., intelligence scores) while considering the relationships between the variables (i.e., EEG channels; Grootswagers et al., 2017). The data-driven nature of MVPA algorithms is particularly advantageous for exploratory research approaches, as they do not require the formulation of a priori hypotheses (Brunton & Beyeler, 2019; Maass et al., 2018). While such algorithms have been employed to examine the predictability of Big Five personality traits from resting-state EEG data (Fruehlinger et al., 2024; Jach et al., 2020; Korjus et al., 2015; Pacheco et al., 2024), analogous studies for fluid and crystallized intelligence measures are lacking. Thus, using MVPA algorithms provides a powerful tool to explore and discern links between brain activity and cognitive abilities.

Addressing the limitations of previous work and harnessing the advantages of MVPA algorithms, this study aimed to clarify the association between spectral EEG power and cognitive abilities. To this end, we preregistered an MVPA (plus various additional analyses) of a large sample of over 800 participants from the CoScience Personality Project (Paul et al., 2022b) while controlling for potential state sleepiness effects. We estimated the decoding performance for fluid and crystallized intelligence from spectral EEG power between 0.5 and 30 Hz. By examining four different conditions (resting-states with eyes open and closed before and after unrelated tasks), we evaluated the stability of potential effects. We defined an effect as meaningful if the decoding performance reached *r* = 0.20 (i.e., a typical effect size in personality psychology; Gignac & Szodorai, 2016) consistently across conditions (Fruehlinger et al., 2024; Jach et al., 2020; Pacheco et al., 2024).

We expected to find a meaningful relationship between resting-state spectral EEG power and fluid and crystallized intelligence scores, as well as state sleepiness. Supplementing the exploratory analyses using MVPA with more targeted a priori defined tests with even higher statistical power, we also examined whether resting-state spectral EEG power and fluid intelligence correlated positively within the alpha band (Doppelmayr et al., 2002; Grandy et al., 2013; Hanslmayr et al., 2005; Jaušovec & Jaušovec, 2000; Klimesch, 1999; Makowski & Troche, 2024; Stankova & Myshkin, 2016; Zoefel et al., 2011) and negatively within the theta band (Klimesch, 1999; Stankova & Myshkin, 2016), as well as whether state sleepiness correlated positively within both frequency bands (Tran et al., 2020). Furthermore, we predicted that state sleepiness would be associated with fluid intelligence, but not, or to a lesser extent, with crystallized intelligence scores (Alhola & Polo-Kantola, 2007; Quigley et al., 2000) and aimed to probe whether any potential associations between resting-state spectral EEG power and both intelligence scores remained robust after controlling for this likely confound. Lastly, we investigated potential sex differences in these associations (Doppelmayr et al., 2002; Jaušovec & Jaušovec, 2005, 2010).

## Methods

### Participants

The CoScience data set includes 819 participants (published studies with this data set: Beauducel et al., 2024; Fruehlinger et al., 2024; Paul et al., 2025). Participants with incomplete data due to problems during data collection and more than four bad channels were removed during preprocessing. Participants were also excluded if their scores of the Intelligence-Structure-Test Revised (I-S-T 2000 R) matrix reasoning or knowledge tests were outside 3.3 standard deviations (*SD*) below the sample mean*.* The final sample consisted of 772 participants between 18 and 30 years old (*M* = 23.39, *SD* = 2.97, 48.77% female). The majority of participants in this sample were European (91.61%), had a high school (66.32%) or university diploma (31.85%), and were university students (91.35%) at the time of data collection (26.4% psychology students). Participants received financial reimbursement or study participation credits. The sample was split into male and female subsets. The male subset included 386 participants and the female subset included 377 participants. There were missing age and gender data from nine participants (1.17%), which were only included in the total sample.

## Materials and measures

This study was preregistered before accessing the data (https://doi.org/10.17605/OSF.IO/JB64Z). The CoScience data set included three resting-state measurements that lasted 8 min. During each measurement, 1-min intervals of open and closed eyes alternated, which were announced by a soft 500-ms acoustic tone. For methodological consistency and in line with our previous analysis of this data set for a different purpose (Fruehlinger et al., 2024), we used the first two resting-state measurements. These resting-state measurements were recorded before and after two unrelated tasks (i.e., Go-NoGo Task & Gambling Task; for more information please refer to Paul et al., 2022b).

Prior to the EEG laboratory session, participants filled out an online questionnaire that contained demographic information and various personality questionnaires that are not included in this study (for an overview see Paul et al., 2022b). At the beginning of the EEG laboratory session, the participants completed 20 items from the matrix reasoning test of the I-S-T 2000 R (Liepmann et al., 2007) within 10 min to assess fluid intelligence. At the end of the laboratory session, the participants answered 84 items from the I-S-T 2000 R’s knowledge test (Liepmann et al., 2007) within 40 min to assess crystallized intelligence. The I-S-T 2000 R is a commonly used intelligence test in Germany. The matrix reasoning and knowledge tests display the strongest loadings on the fluid and crystallized intelligence factors within its standardization sample, which is why they are explicitly described as marker variables for the respective factors (Liepmann et al., 2007).

Self-reported mood for ten different mood states was collected nine times during data collection. Participants indicated on a 7-point Likert scale how much the respective mood represented their current mood. The items were similar to the Profile of Mood States (POMS; Albani et al., 2005 after McNair et al., 1992). We calculated pre- and post-task state sleepiness by averaging the *z*-standardized scores from the tiredness and exhaustion items (pre-task measurement closest to matrix reasoning test and first resting-state measurement, and post-task measurement closest to second resting-state measurement). Both are assigned to the fatigue dimension of the POMS and correlated with *r* = .66, *p* < .001 in the pre-task condition (Spearman-Brown corrected *r* = .79) and *r* = .67, *p* < .001 in the post-task condition (Spearman-Brown corrected *r* = .80).

### EEG recording and preprocessing

EEG was acquired from active or passive gel-filled Ag–AgCl electrodes placed according to the 10–20 system using either Biosemi, BrainAmp, or ActiCHamp systems (for details, see Paul et al., 2022a). The electrodes included 61–64 scalp electrodes, 2 mastoids, and 2–4 electrooculogram electrodes positioned on the external canthi and the sub- and supra-orbit of the eye. EEG recordings were grounded to AFz or FPz (where necessary) and referenced to Cz, FCz, or common mode sense and driven right leg electrodes (CMS/DRL). The sampling rate was either 512 Hz or 500 Hz.

The fully automatized preprocessing pipeline was implemented in Matlab 2023a (The MathWorks Inc., 2023) and EEGLAB 2023.0 (Delorme & Makeig, 2004) and is identical to our previous pipeline (Fruehlinger et al., 2024) that was based on the methods described in Jach et al. (2020). The data were referenced to Cz, as the online references differed across CoScience research sites, and filtered with basic finite impulse response (FIR) filters (0.5 Hz high-pass, 30 Hz low-pass). Flat or artifact-ridden channels were removed with FASTER (Nolan et al., 2010), based on deviations of variance, amplitude, and channel deviation (using the default threshold of *z* = 3). The data set was excluded if more than four channels were labeled bad (61 data sets in total; mean of excluded channels: 2.22). Next, the data were separated into 1-min epochs based on the onset of the acoustic tone. The epochs were shortly delayed to attenuate the potential effects of the soft acoustic signal. Bad segments within the epochs were identified and removed with artifact subspace reconstruction (ASR; on a copy of 1 Hz high-pass filtered data). We applied independent component analysis (ICA) infomax and principal component analysis (PCA). ICs comprising eye components with a probability of > 70% and brain components with < 70% were identified and removed using IClabel (mean: 1.55 for both conditions; Pion-Tonachini et al., 2019). The data were segmented into 2-s epochs with an overlap of 1 s and tapered with a Hamming window. We applied the following artifact criteria to remove epochs: (1) voltage difference > 50 µV every two sampling points (i.e., 1 ms), (2) voltage difference > 200 µV in a 200-ms interval, and (3) voltage difference < 0.5 µV between the minimum and the maximum voltage in a 100-ms interval. The preprocessed data were converted into the frequency domain by applying the fast Fourier Transformation (FFT) with a 0.5 Hz resolution. The power spectra of the total signal ranging from 0.5 Hz to 30 Hz were used in subsequent MVPA. As reported in our previous study, the median reliability was *r* = .81 for the pre- and post-task conditions with eyes open and *r* = .87 with eyes closed (Fruehlinger et al., 2024).

Additionally, the total signal was disentangled into its periodic and aperiodic signal components using the “fitting oscillations & one over f” (FOOOF) toolbox (version 1.0.0; Donoghue et al., 2020). This pipeline was run in Spyder 5.2.2 using Python 3.9.12. The settings for the algorithm were similar to Pacheco et al. (2024) and were set as *peak width limits: [1, 12]*, and *aperiodic mode: fixed,* otherwise default settings (*maximum number of peaks: inf., minimum peak height: 0, peak threshold: 2.0*). The algorithm parameterized the power spectra with a 0.5 Hz resolution for the periodic signal component (i.e., aperiodic signal components have been removed from the total signal) and calculated the individual aperiodic offset and exponent parameters.

### MVPA

The decoding approach is identical to our previous study (Fruehlinger et al., 2024). We used the Decision Decoding Toolbox (DDTBox; Bode et al., 2019) and applied support vector regressions (SVR) with a linear kernel. The feature space contained the frequency spectra from 30 frequency bins across 59 EEG channels (i.e., number of consistent channels across research sites). Participants’ intelligence and sleepiness scores were predicted from EEG spectral power within 1 Hz frequency bins between 0.5 and 30 Hz. The frequency bins were calculated by averaging sets of 0.5 Hz. For example, the 1 Hz frequency bin included the data from 0.5 Hz and 1 Hz from the FFT. The SVR algorithm was performed in LIBSVM (Chang & Lin, 2011), which was implemented in the DDTBox (Bode et al., 2019). The three-dimensional input matrix included spectral power values between 0.5 and 30 Hz for each EEG channel and participant. Separate SVR analyses were performed for each signal type (total, periodic signal, and aperiodic parameters), condition (eyes open or closed before and after unrelated tasks), intelligence, and sleepiness measure, for the total and the male and female subsamples.

We applied a tenfold cross-validation for all analyses and repeated this procedure 10 times to account for false-positive results and diminish random fluctuations in prediction performance. The resulting decoding performance was averaged over analysis repetitions and Fisher-Z-transformed. This provided full predictive accuracy between the frequency bins and the intelligence as well as sleepiness scores.

### Statistical analysis

#### MVPA decoding

Similar to our previous study (Fruehlinger et al., 2024) and others (Jach et al., 2020; Pacheco et al., 2024), we defined an effect as meaningful if the decoding performance reached *r* = .20 (i.e., a typical effect size in personality psychology; Gignac & Szodorai, 2016) consistently across conditions.

To perform permutation tests, intelligence or sleepiness scores were randomly assigned to an EEG data set and SVR was applied to this data (where no correlations were to be expected between the EEG data and the intelligence or sleepiness scores). This procedure was repeated 1,000 times per regression analysis. With this large number of iterations, we established the distribution under the null hypothesis. This allowed the estimation of the *p*-values. This was done by comparing the actual decoding performance for each frequency bin to the null distribution of the permutation.

To estimate potential gender differences, we focused on frequency bin clusters with meaningful results in either male or female subsamples. A cluster was defined as a group of adjacent meaningful frequency bins. We calculated the difference in the mean decoding performance between men and women for these frequency clusters and tested for significance using *z*-tests. We corrected for multiple comparisons by applying Bonferroni-Holm correction.

#### Spearman correlations

We calculated Spearman correlations between both fluid as well as crystallized intelligence scores and state sleepiness scores. Additionally, we calculated correlations between fluid intelligence and average EEG spectral power (total and periodic) in the theta (i.e., 4–8 Hz) and alpha frequency (i.e., 8.5–13 Hz) bands across conditions and electrodes. For state sleepiness, we also calculated correlations but averaged EEG spectral power values in the theta and alpha frequency bands across electrodes within pre- and post-task conditions. This ensured that the EEG power values correspond to the timepoint of the state sleepiness measurements. We applied Bonferroni-Holm correction for six tests (correlations between fluid intelligence and mean theta and alpha power; correlations between pre/post sleepiness scores with mean pre/post theta, and alpha power) to adjust for *p* < .05 within each signal type. These correlations were computed in R (version 4.4.2; R Core Team, 2024) using RStudio (version 2024.12.0; Posit team, 2024). The remaining analyses were conducted in Matlab 2023a (The MathWorks Inc., 2023).

Finally, we estimated the robustness of potential associations between resting-state spectral EEG power and both intelligence scores after controlling for state sleepiness by calculating post hoc partial Spearman correlations within each meaningful frequency bin and EEG channel. This allowed us to determine the direction of the effect.

#### Exploratory correlations

To investigate the spatial distribution of the associations between brain power and intelligence as well as sleepiness scores, we calculated Spearman correlations between the mean power of each frequency band at each electrode and the behavioral scores. We corrected for multiple comparisons using false discovery rate (FDR) for four frequency bands, four conditions, and 59 electrodes for each score at a significance level of α = .05. For these exploratory analyses FDR was more appropriate than Bonferroni-Holm correction that we applied in our preregistered analyses. This correction would be too conservative given the high number of tests, the investigation of the spatial distribution, and the spatial dependency of the data in this analysis.

### Replication analysis

In an additional analysis, we originally aimed to replicate potential meaningful results between resting-state EEG power and fluid intelligence scores by applying the same analysis approach to the data set described in Ociepka et al. (2022). However, because we did not find any meaningful associations, we did not conduct this analysis.

### Use of generative AI and AI-assisted technologies in the writing process

During the preparation of this work, the authors used ChatGPT-4o and ChatGPT-4o mini in order to improve readability and language. After using this tool, the authors reviewed and edited the content as needed and take full responsibility for the content of the publication.

## Results

### Descriptive statistics and preliminary analysis of intelligence and sleepiness scores

Table 1 shows means, standard deviations, McDonald’s omegas, and correlations between intelligence and sleepiness scores. Figure S1 in the supplementary material shows the distribution of the behavioral variables graphically. The internal consistency was high (ω ≥ .83) except for the fluid intelligence test. This is likely partly due a somewhat lower variance in the I-S-T 2000 R’s matrix reasoning test compared to the one reported in the manual for a representative sample (var_CoS_ = 7.02, var_Manual_ = 11.16; *F*(477, 771) = 1.59, *p* < .001; Liepmann et al., 2007). Note, however, that the internal consistency in our sample corresponds to that in other student samples (Bühner et al., 2006) and that the reduction of variance can only account for a relatively modest reduction of potential associations (e.g., from *r* = .20 to *r* = .13, based on Thorndike Case 2 and the formula provided by Stauffer & Mendoza, 2001). Post-task sleepiness was significantly higher (*M* = 0.33, *SD* = 0.94) than pre-task sleepiness (*M* =  −0.36, *SD* = 0.74), *t*(771) = 23.38, *p* < .001, *d* = 0.80, 95% confidence interval (CI) [0.72, 0.87].

**Table 1:** Means, standard deviations, and McDonald’s omega of the behavioral variables

Variable	*M*	*SD*	ω
Fluid intelligence	10.99	2.65	.57
Crystallized intelligence	50.35	9.35	.86
Pre-task sleepiness	−0.36	0.74	.83
Post-task sleepiness	0.33	0.94	.84

*M* mean, *SD* standard deviation, *ω* McDonald’s omega, *N = *772

Next, we calculated Spearman correlations to estimate the relationship between both fluid and crystallized intelligence scores, and state sleepiness scores. As expected, the association between state sleepiness scores was numerically stronger for fluid intelligence (*rho* =  −.07, 95% CI [−.13, −.01], *p*_*adj*_ = .073) than for crystallized intelligence (*rho* =  −.03, 95% CI [−.09, .03], *p*_*adj*_ = .23), although both were very small and nonsignificant after correction for the two comparisons.

### Intelligence

#### MVPA results

In the total signal, the decoding performance for fluid and crystallized intelligence scores within the full sample did not surpass the pre-defined threshold of *r* = .20 in any frequency bin or condition. The highest decoding performance for fluid intelligence scores was at 5 Hz in the post-task eyes open condition, *r* = .11, 95% CI [.10, .13]. For crystallized intelligence, the highest decoding performance was found in the 13 Hz frequency bin for the pre-task eyes open condition, *r* = .14, 95% CI [.11, .18]. Supplementing the preregistered analyses, we used the permutation data to perform an exploratory cluster correction for *p* = .01 with a minimum cluster size of two frequency bins. In these exploratory analyses, no significant clusters were detected in the total signal for either intelligence score in the exploratory cluster correction (Fig. S2 in the supplementary material).

**Fig. 1:**
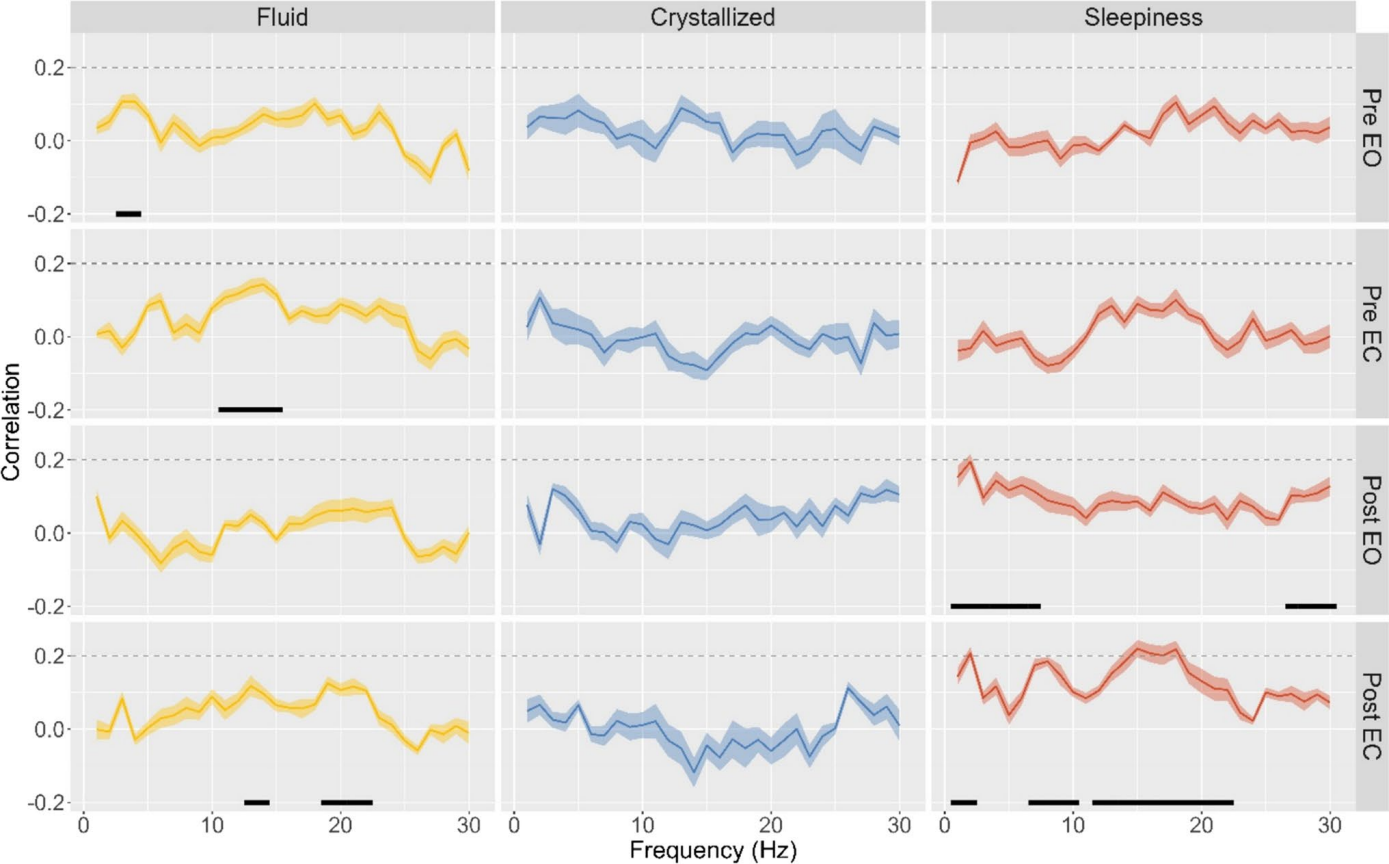
Mean decoding performance for fluid and crystallized intelligence, and state sleepiness scores in the periodic signal. Shaded areas indicate 95% confidence interval around the correlation coefficient. Black lines indicate significant clusters with *p*_*adj*_ < .01

For the periodic signal (Fig. 1), the decoding performance for both intelligence scores remained below *r* = .20 (fluid intelligence: highest decoding performance at 14 Hz in the pre-task eyes closed condition, *r* = .14, 95% CI [.12, .16]; crystallized intelligence: peak decoding performance at 3 Hz in the post-task eyes open condition, *r* = .12, 95% CI [.11, .14]). However, the exploratory analyses indicated significant clusters for fluid intelligence in the pre-task condition for 3 and 4 Hz and between 11 and 15 Hz, with eyes open and closed respectively, and in the post-task condition with eyes closed for 13 and 14 Hz as well as 19–22 Hz.

Lastly, the aperiodic parameters were consistently below *r* = .20 (Table S1 in the supplementary material). Also note, that lowering the pre-defined threshold of *r* = .20 to *r* = .13 to account for the effect of a somewhat reduced fluid intelligence variance in our student sample (see initial paragraph of the Results) did not alter the conclusions: Neither of the intelligence measures demonstrated a decoding performance of at least *r* = .13 across the four conditions in either of the analyses (total signal, periodic signal, and aperiodic signal).

#### Spearman correlation results

Spearman correlations between the mean resting-state EEG theta (4–8 Hz) and alpha power (8.5–13 Hz) and fluid intelligence in the total signal were not significant (theta: *rho* =  −.03, 95% CI [−.09, .04], *p*_*adj*_ = 1.00; alpha: *rho* = .00, 95% CI [−.07, .06], *p*_*adj*_ = 1.00).

This was also the case within the periodic signal (theta: *rho* =  −.03, 95% CI [−.09, .03], *p*_*adj*_ = 1.00; alpha: *rho* = .01, 95% CI [−.05, .07], *p*_*adj*_ = 1.00).

#### Exploratory correlation results

Because we did not find any meaningful results regarding the decoding performance of fluid and crystallized intelligence, exploratory partial Spearman correlations were calculated between brain power (in four frequency bands, at 59 electrodes, in four recording conditions) and intelligence scores while controlling for sleepiness. After correction for multiple comparisons using FDR no significant correlations were observed.

#### Gender effects on the decoding of intelligence

We could not find any meaningful results for the male and female subsamples in either spectral signal type. Therefore, we did not calculate the difference in mean decoding performance between men and women. Figures S3–S6 in the supplementary material show that there are significant frequency bins based on the exploratory cluster correction that differ between men and women. However, these bins were not consistent across conditions, therefore we did not consider them reliable as preregistered. Notably, the decoding performance for both aperiodic parameters surpassed the threshold regarding crystallized intelligence within the male subsample, but only for the post-task condition with eyes closed (exponent: *r* = .21, 95% CI [.18, .23], offset: *r* = .24, 95% CI [.21, .27]; Table S1 the supplementary material).

### State sleepiness

#### MVPA results

In the total signal, the highest decoding performance for state sleepiness was *r* = .19, 95% CI [.16, .22] for 13 Hz in the post-task eyes open condition. We found significant decoding clusters for state sleepiness in the pre-task condition with eyes open for 12 and 13 Hz, in the post-task condition with eyes open for 5–6 Hz and 12–16 Hz, as well as in the post-task condition with eyes closed for 5–6 Hz (Fig. S2 in the supplementary material).

When we repeated this analysis for the periodic signal (Fig. 1), sleepiness in the post-task condition with eyes closed was predicted with *r* > .20 for frequencies 2 Hz and 15–18 Hz. There were significant clusters in the post-task condition between 1–7 Hz and 27–30 Hz with eyes open as well as 1–2 Hz, 7–10 Hz, and 12–22 Hz with eyes closed. The decoding performance of the aperiodic parameters did not reach *r* = .20 in any condition (Table S1 in the supplementary material).^1^

#### Spearman correlation results

Regarding the specific associations between spectral power and state sleepiness in the total signal, we found a significant correlation between mean theta power and sleepiness scores in the post-task condition, *rho* = .09, 95% CI [.03, .15], *p*_*adj*_ = .039. The remaining correlations were not significant (theta: *rho* = .00, 95% CI [−.06, .06], *p*_*adj*_ = 1.00; alpha: *rho* =  −.04, 95% CI [−.10, .02], *p*_*adj*_ = 1.00, in the pre-task condition; alpha: *rho* = .00, 95% CI [−.06, .06], *p*_*adj*_ = 1.00, in the post-task condition).

There were no significant correlations within the periodic signal (theta: *rho* =  −.02, 95% CI [−.08, .04], *p*_*adj*_ = 1.00; alpha: *rho* =  −.06, 95% CI [−.12, .00], *p*_*adj*_ = 1.00, in the pre-task condition; theta: *rho* = .02, 95% CI [−.05, .08], *p*_*adj*_ = 1.00; alpha: *rho* =  −.03, 95% CI [−.09, .03], *p*_*adj*_ = 1.00, in the post-task condition).

#### Exploratory correlation results

**Fig. 2:**
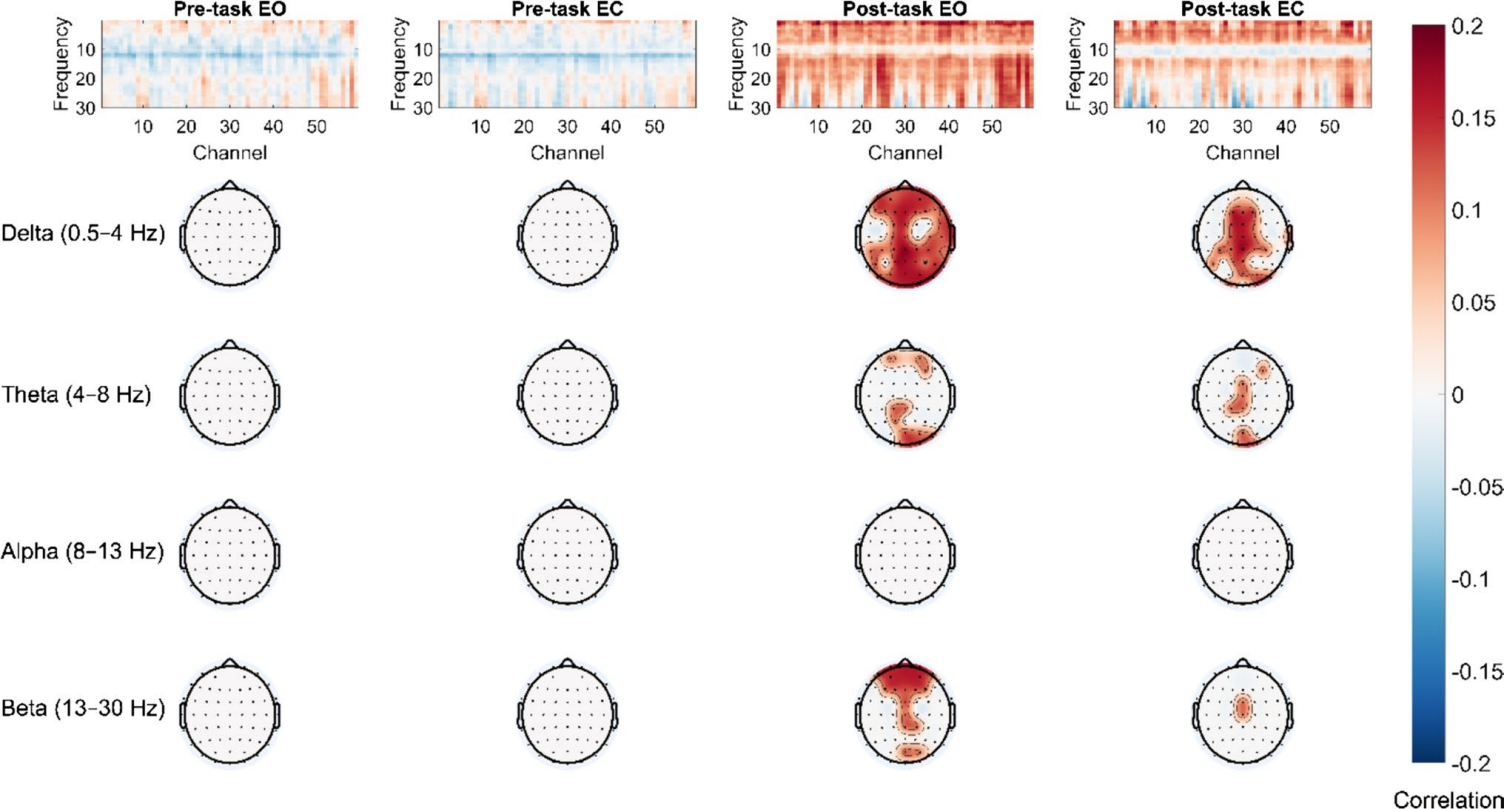
Correlations between mean spectral power for each frequency and sleepiness scores for each condition. Topographical plots show significant correlations after correcting for multiple comparisons using FDR in specific frequency bands

**Fig. 3:**
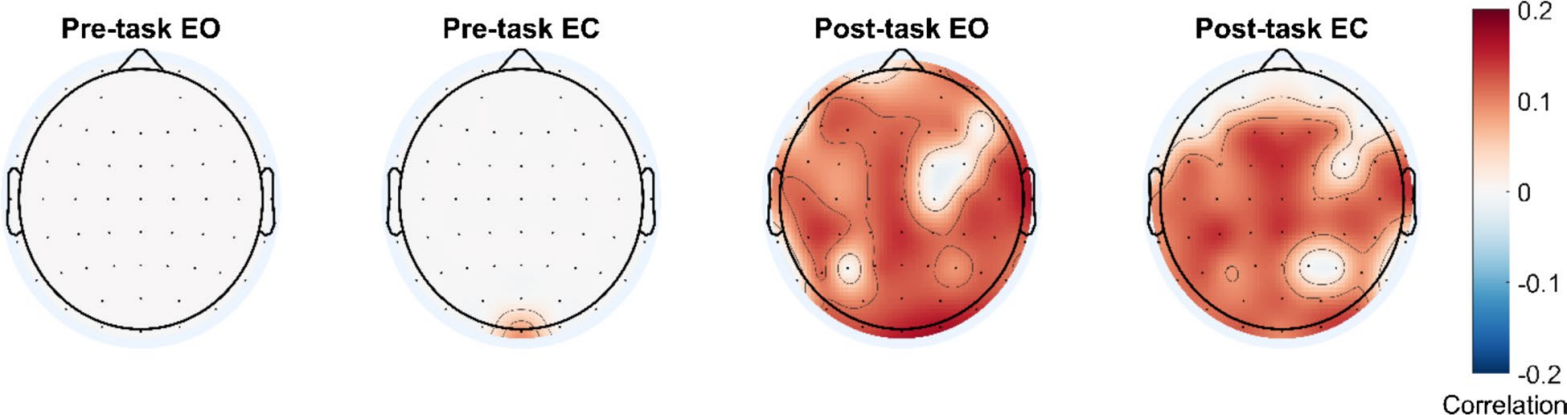
Significant correlations between aperiodic offset parameter and sleepiness scores for each condition after correcting for multiple comparisons using FDR

We found significant correlations between sleepiness scores and delta (0.5–4 Hz) as well as theta (4–8 Hz) frequencies in the total signal within both post-task conditions. These correlations were positive and mainly observed at frontal, central, and posterior electrode positions (highest correlation: *r* = .20 at FCz for 1.5 Hz in the post-task eyes closed condition; Fig. 2). In addition, the aperiodic offset parameter also significantly correlated with sleepiness scores in the post-task conditions (highest correlation: *r* = .17 at O2 in the post-task eyes open condition; Fig. 3). There were no significant correlations in the periodic signal for any frequency band or condition.

## Discussion

In this study, we used the CoScience data set (Paul et al., 2022b) to investigate the predictability of fluid and crystallized intelligence from resting-state EEG power. Despite our large sample size (*N* = 772), neither the decoding approach using MVPA on the total EEG signal and its periodic and aperiodic signal components nor the a priori defined correlations based on previous reports (Doppelmayr et al., 2002; Grandy et al., 2013; Hanslmayr et al., 2005; Jaušovec & Jaušovec, 2000; Klimesch, 1999; Makowski & Troche, 2024; Stankova & Myshkin, 2016, 2016; Zoefel et al., 2011) revealed any meaningful associations between EEG spectral power and intelligence. Supplementary exploratory analyses showed associations with fluid intelligence in distinct, albeit only partially consistent, frequency clusters (pre-task eyes open: 3–4 Hz; pre-task eyes closed: 11–15 Hz; post-task condition eyes closed: 13–14 Hz and 19–22 Hz). The analysis of gender-specific effects indicated that only for male participants, specifically the aperiodic parameters in the post-task condition with eyes closed, were associated with crystallized intelligence. Analyses were repeated to predict state sleepiness scores aiming to control for potential confounding effects that could obscure the associations between intelligence scores and resting-state EEG power. Our MVPA approach revealed decoding performances of *r* > .20 for frequencies 2 Hz and 15–18 Hz for the periodic signal (significant clusters in the post-task eyes open: 1–7 Hz and 27–30 Hz; post-task eyes closed: 1–2 Hz, 7–10 Hz, and 12–22 Hz). The Spearman correlation between mean theta power in the total signal and state sleepiness was significant in the post-task condition. Exploratory analyses found significant correlations between state sleepiness and delta (0.5–4 Hz) and theta (4–8 Hz) frequencies in the total signal as well as significant correlations with the aperiodic offset parameter within both post-task conditions. The following sections contextualize the findings within the existing literature and make suggestions for further research.

While we did not identify any reliable associations between intelligence and resting-state EEG power, these findings are not surprising and align with the broader body of previous research. Although earlier studies occasionally reported associations between alpha or theta power and cognitive abilities (Doppelmayr et al., 2002; Grandy et al., 2013; Jaušovec & Jaušovec, 2000; Jaušovec & Jaušovec, 2000; Klimesch, 1999; Klimesch, 1999; Makowski & Troche, 2024; Stankova & Myshkin, 2016; Stankova & Myshkin, 2016; Thatcher et al., 2005), these results are inconsistent and limited by small sample sizes and methodological variability. Our study, in contrast, was preregistered, employed stringent analytical methods, and utilized a large sample size, providing a more robust evaluation of these associations. These strengths bolster the reliability of our findings and suggest the lack of substantial correlations between resting EEG power and cognitive abilities. Moreover, these null-findings align with other studies with at least moderate sample sizes (*N* > 153), which also did not find such associations (Grandy et al., 2013; Ociepka et al., 2022). Additionally, when accounting for potential confounding effects of state sleepiness (Alhola & Polo-Kantola, 2007; Quigley et al., 2000) the results did not change, which further reinforces the robustness of our conclusions.

It is generally assumed that individual differences in cognition, behavior, and motivation should be associated with, but not reducible to, differences in brain activity (DeYoung, 2010; DeYoung & Gray, 2009; Wacker & Paul, 2022). However, multifaceted traits, like intelligence, may be too complexly integrated into the brain and potentially superimposed by transient states (e.g., sleepiness) to be captured by our decoding approach. Previously reported links between state sleepiness and brain activity underscores this assumption (Tran et al., 2020). In general, our findings align with the meta-analytic conclusions of Tran et al. (2020), which identified increased theta power—particularly at frontal, central, and posterior channel positions—as a biomarker for state sleepiness. However, we found this association only in the post-task conditions, which might be owing to a significantly lower sleepiness variance in the pre-task condition. Additionally, we found significant correlations between post-task sleepiness scores and spectral power within the delta band. Indeed, studies investigating the effects of sleepiness (due to sleep deprivation or driver fatigue) also reported increased delta activity (De Gennaro et al., 2007; Lal & Craig, 2002; Tinguely et al., 2006). Overall, the consistency of our results with these patterns attests to a sufficient quality of the data set and demonstrates the effectiveness of the MVPA methodology in capturing meaningful neural correlates. Aligning with the reasoning of Chatburn et al. (2024), the correlations observed were primarily driven by the aperiodic offset parameter, and not the periodic signal. This indicates that sleepiness might involve a general shift in baseline neural excitability (as indexed by the aperiodic EEG component; Donoghue et al., 2020) rather than a modulation of specific rhythmic activities. In fact, previous studies found an increase in neural excitation with prolonged wakefulness (Huber et al., 2013; Kuhn et al., 2016). These results further highlight the utility of this decomposition approach, reinforcing its importance in uncovering meaningful and distinct electrophysiological patterns.

One reason we may not have identified significant associations with intelligence is that our preregistered threshold of *r* = .20, which was based on previous research (Fruehlinger et al., 2024; Jach et al., 2020), might have been overly conservative. As outlined above, the exploratory cluster correction analyses showed significant (yet inconsistent) clusters for adjacent frequency bins for fluid intelligence in the periodic signal. Indeed, these clusters are decoded with *r* ≈ .10, which indicates that significant effects might be smaller than *r* = .20. However, the meaningfulness of such a small effect continues to be a topic of debate within the field (Funder & Ozer, 2019; Nickel, 2020). Note that although potentially interesting, these exploratory results should be considered with caution until replicated in a sufficiently large independent sample along with a thorough preregistration of all analysis steps.

Another explanation for the lack of significant findings may lie in the limitations of analyzing frequency bands at rest, which might not adequately capture the neural correlates of cognitive abilities (for reviews see Hilger et al., 2022; Jaušovec, 2019). First, resting-state EEG may not be as informative as task-related EEG data (DeYoung et al., 2025). Second, the P-FIT, which posits that individual differences in intelligence arise from the efficient integration of information across parietal and frontal brain regions, highlights the importance of brain network variations (Basten et al., 2015; Jung & Haier, 2007; Santarnecchi et al., 2017). Thus, complexity or connectivity measures of the EEG signal may be better predictors of cognitive abilities. Following this suggestion, Dreszer et al. (2020) applied a multivariate multiscale sample entropy (mMSE) analysis to resting EEG data and found that spatiotemporal complexity patterns significantly predicted fluid intelligence. Thiele et al. (2023) expanded this work by analyzing multiple entropy measures and microstate features separately, as well as a combined multimodal model. This multimodal model predicted intelligence scores both within a discovery and an independent replication sample. Similarly, Santarnecchi et al. (2017) discovered an inverse correlation between fluid intelligence and characteristics of certain microstates. The results of these three studies are promising given their—for EEG studies—relatively large sample size (*N* = 74–144). They suggest that neural correlates of intelligence exist, but they are less obvious and require more sophisticated methods to uncover. However, independent replication studies, ideally with large samples and a detailed preregistration of all analysis steps, are needed to assess the robustness of these results as well.

Finally, another limitation of our study lies in the composition of our sample, which primarily consisted of young university students. This homogeneity resulted in a somewhat reduced variability in cognitive abilities and relatively low internal consistency of the fluid intelligence measure (compared to the I-S-T 2000 R’s manual), and consequently, potentially smaller associations and, thus, lower statistical power to detect them. Whereas corrections for restriction of variance and/or attenuation only suggest modest reductions of potential effect sizes (and thus statistical power) due to this issue, the generalizability of our results is still limited given our Western, educated, industrialized, rich, and democratic (WEIRD; Henrich et al., 2010) sample underscoring the need for future research to incorporate more diverse samples with broader age and ability ranges to improve generalizability and enhance the ability to detect subtle effects.

Despite these limitations, our study is characterized by several important strengths. The use of a large, preregistered data set with stringent analytical methods ensures that our results are robust and transparent, setting a benchmark for future research. The integration of MVPA to analyze periodic and aperiodic components of the EEG signal represents a novel approach that advances the methodological toolkit available for exploring complex neural correlates of cognitive abilities. Moreover, the null findings are valuable in refining the theoretical framework surrounding intelligence and neural activity. By failing to identify meaningful associations in such a rigorous study, we provide evidence that challenges simplistic models linking resting-state EEG power to intelligence. These findings encourage a shift toward more sophisticated measures of brain activity, such as network dynamics or multiscale analyses, and help delineate the limits of current methodologies.

## Conclusions

The present study investigated the predictability of fluid and crystallized intelligence scores from resting-state EEG data. We could not find meaningful results in either EEG signal type based on our thresholds. Despite these null results, the replication of previously reported links between resting-state EEG power and state sleepiness attests to a sufficient quality of the CoScience EEG data (Paul et al., 2022b). Additionally, the significant correlation between state sleepiness and the aperiodic offset parameter highlights the potential of aperiodic EEG features as biomarkers for physiological states. Future studies should explore alternative analysis methods and rigorously preregister all analysis steps to uncover the neural correlates of intelligence.

## Electronic supplementary material

Below is the link to the electronic supplementary material.

Supplementary file1 (DOCX 1974 kb)

## Data Availability

Due to the specifications of the CoScience data set, we cannot share the data openly. Data are therefore available for reproduction tests by requesting it from the authors. For other uses, data are available upon preregistration (see Paul et al., 2022b).

## Appendix 1

### Preregistration Deviations

**Table 2:** Preregistration deviations

Deviations					
No		Details	Original wording	Deviation description	Reader impact
1	Type	Data preparation	In the last step, we will apply all the analyses for the periodic and aperiodic power spectrum, and the aperiodic parameters where appropriate	Based on a reviewer’s comment on Fruehlinger et al. (2024), we did not repeat the analysis for the aperiodic power spectrum, but only for the aperiodic parameters	This deviation has no impact on the interpretation of the results
	Reason	Update/previous mistake			
	Timing	After data access			
2	Type	Analytic approach	For Hypothesis 2, we will calculate Spearman correlations between the average spectral power in the respective frequency bands (across conditions) and both fluid intelligence and sleepiness scores. We will apply Bonferroni-Holm correction for four tests to adjust for *p* < .05	Regarding the correlations between the average spectral power in the specific frequency bands and sleepiness scores, we decided to average the power values within pre- and post-task conditions instead of across conditions. We applied Bonferroni-Holm correction for six instead of four tests to adjust for *p* < .05	This deviation may affect the readers’ interpretation of the results. This deviation was necessary due to the significantly lower variance of pre-task state sleepiness. It further ensured that the EEG power values correspond to the timepoint of the state sleepiness measurement, which provides a clearer picture of the correlational results
	Reason	Preregistered plan inappropriate because of data			
	Timing	After results were known			
Unregistered Steps					
No		Details	Original wording	Unregistered step description	Reader impact
1	Type	Inferential criteria	We will only report the decoding performance and no *p*-values. Thus, correction for multiple comparisons is not necessary	We use the data from the permutation analysis to perform an exploratory cluster correction	This unregistered step affects the readers’ interpretation of the decoding results because results that are below the a priori defined threshold of *r* = .20 and thus considered not meaningful, are potentially significant. Importantly, this cluster correction is labeled “exploratory” throughout the manuscript, which helps the reader to differentiate between preregistered and additional exploratory analyses
	Timing	After results were known			

## Footnotes

1 To control for potential between-lab effects, we exploratively mean-centered the data by site for every electrode, frequency bin, and condition and repeated the MVPA (see also Fruehlinger et al., 2024). The decoding results were similar to the ones reported in the main text and did not change the interpretation.

## Code availability

All scripts are openly available on the Open Science Framework (OSF) platform (https://osf.io/g89b2/) and GitHub (https://github.com/christoph1212/MVPA_IQ).

## Declarations

### Open Practices

This study was preregistered before accessing the data (https://doi.org/10.17605/OSF.IO/JB64Z). We have minor deviations from our preregistration to disclose (i.e., exploratory cluster analysis, number of Spearman correlations between average spectral power and sleepiness scores; see Table 2 in the Appendix; Willroth & Atherton, 2024).
