# Supplementary material for: Sleepiness but neither fluid nor crystallized intelligence can be predicted from resting-state electroencephalography – Evidence from the large scale CoScience EEG-Personality Project
Source: Cogn Affect Behav Neurosci. 2025 Jul 1;25(6):1737–50. doi: 10.3758/s13415-025-01323-y (PMC12615557; doi:10.3758/s13415-025-01323-y)
Supplement: Supplementary file 1 — Supplementary file1 (DOCX 1974 kb) [file 13415_2025_1323_MOESM1_ESM.docx]

Supplementary Material: Sleepiness but Neither Fluid nor Crystallized Intelligence can be Predicted From Resting-State EEG – Evidence From the Large Scale CoScience EEG-Personality Project

Christoph Fruehlinger^1^, Katharina Paul^1^, Corinna Kührt² & Jan Wacker^1^

^1^Department of Differential Psychology and Psychological Assessment, Institute of Psychology, University of Hamburg, Germany

²Faculty of Psychology, Technische Universität Dresden, Dresden, Germany

**List of Tables**

[**Table S1.** Mean decoding performance for aperiodic exponent and offset parameters for each variable, condition, and sample 2](#_Toc182399097)

**List of Figures**

[**Figure S1.** Distributions for all behavioral scores 3](#_Toc196230295)

[**Figure S2.** Mean decoding performance for fluid and crystallized intelligence, and state sleepiness scores in the total signal 4](#_Toc196230296)

[**Figure S3.** Mean decoding performance for fluid and crystallized intelligence, and each condition in the total signal for the male subsample 5](#_Toc196230297)

[**Figure S4.** Mean decoding performance for fluid and crystallized intelligence, and each condition in the total signal for the female subsample 6](#_Toc196230298)

[**Figure S5.** Mean decoding performance for fluid and crystallized intelligence, and each condition in the periodic signal for the male subsample 7](#_Toc196230299)

[**Figure S6.** Mean decoding performance for fluid and crystallized intelligence, and each condition in the periodic signal for the female subsample 8](#_Toc196230300)

**Table S1.** Mean decoding performance for aperiodic exponent and offset parameters for each variable, condition, and sample.

|  | Exponent | | | Offset | | |
| --- | --- | --- | --- | --- | --- | --- |
|  | Fluid | Crystallized | Sleepiness | Fluid | Crystallized | Sleepiness |
| Full Sample | | | | | | |
| Pre EO | −.01 | .08 | .12 | .02 | .15 | .01 |
| Pre EC | −.01 | .05 | .06 | −.02 | .05 | .05 |
| Post EO | .01 | .01 | .06 | .00 | .04 | .07 |
| Post EC | .03 | .10 | .01 | .02 | .11 | .10 |
| Male Sample | | | | | | |
| Pre EO | −.01 | .15 | – | .00 | .14 | – |
| Pre EC | −.12 | .18 | – | −.07 | .11 | – |
| Post EO | −.04 | .06 | – | .02 | .12 | – |
| Post EC | −.03 | **.21** | – | .00 | **.24** | – |
| Female Sample | | | | | | |
| Pre EO | −01 | −.13 | – | .01 | .04 | – |
| Pre EC | −.05 | −.03 | – | −.02 | .03 | – |
| Post EO | .03 | −.07 | – | −.04 | .00 | – |
| Post EC | .06 | −.03 | – | .03 | .04 | – |

*Note*. Decoding performances of *r* ≥ .20 are written in bold. Pre = Pre-Task, Post = Post-Task, EO = Eyes-Open, EC = Eyes-Closed.


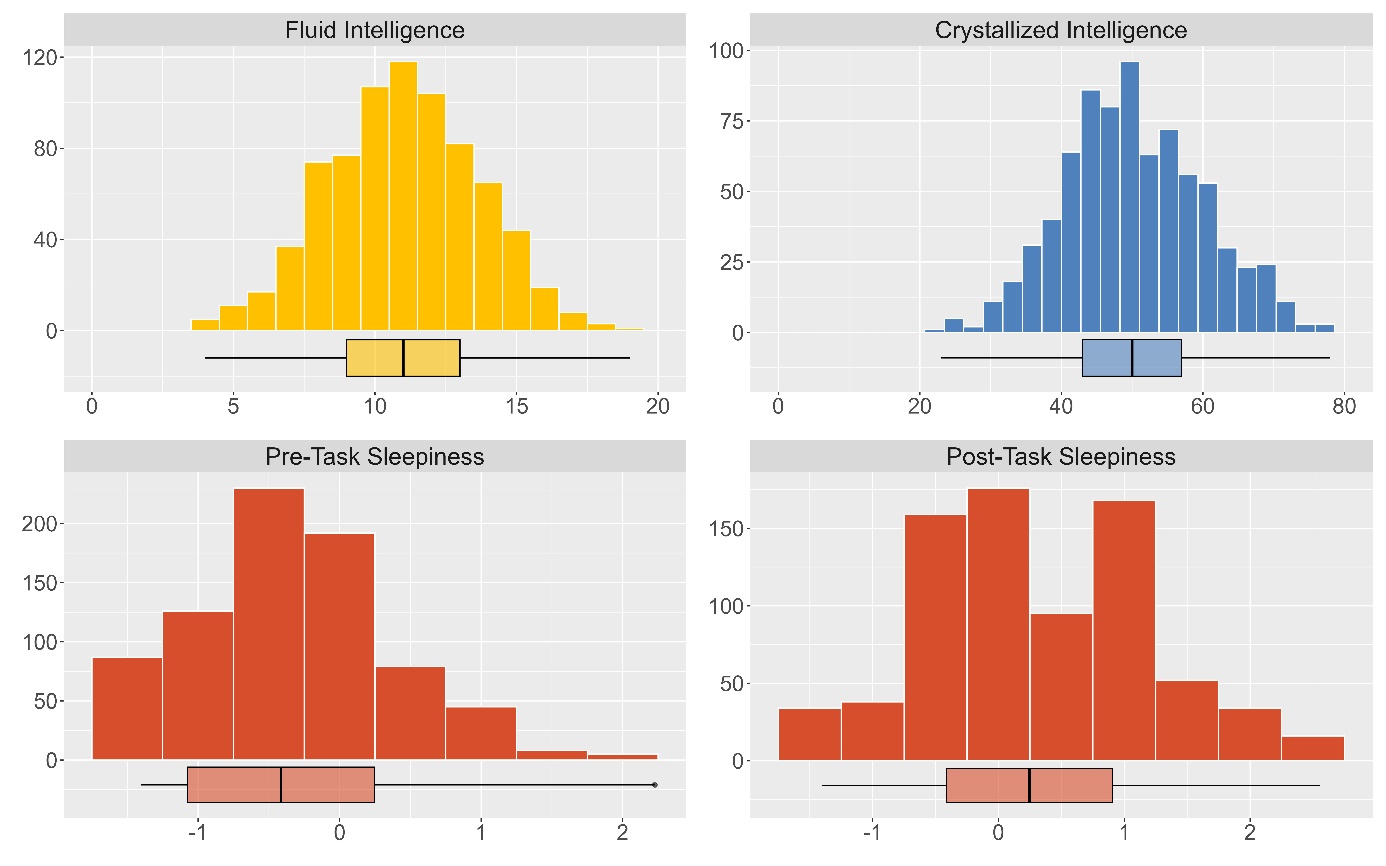


**Figure S1.** Distributions for all behavioral scores. X-axes for fluid and crystallized intelligence reflect number of items in each test.


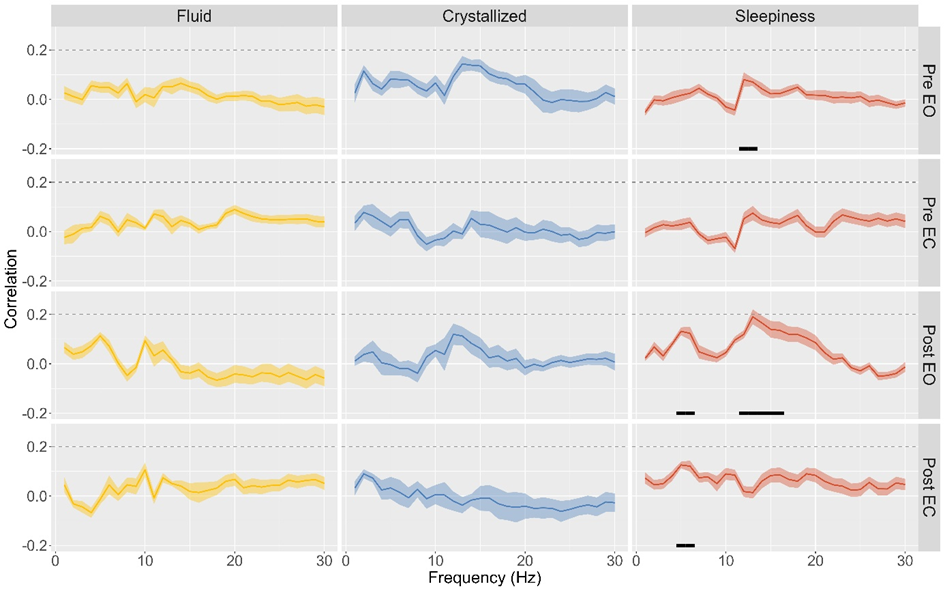


**Figure S2.** Mean decoding performance for fluid and crystallized intelligence, and state sleepiness scores in the total signal. Shaded areas indicate 95% confidence interval around the correlation coefficient. Black lines indicate significant clusters with p < .01. The decoding performance did not consistently surpass the predefined threshold. Pre = Pre-Task, Post = Post-Task, EO = Eyes-Open, EC = Eyes-Closed.


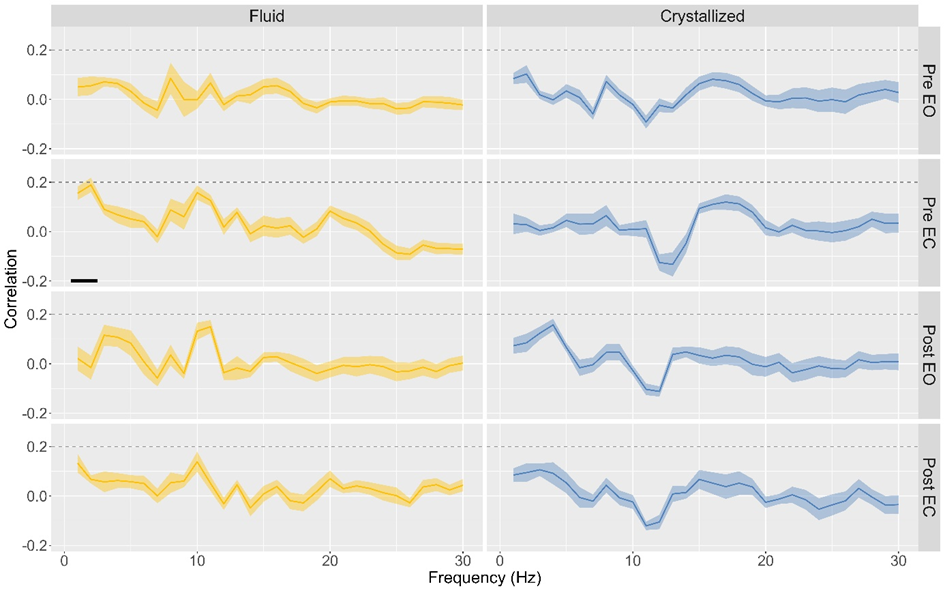


**Figure S3.** Mean decoding performance for fluid and crystallized intelligence, and each condition in the total signal for the male subsample. Shaded areas indicate 95% confidence interval around the correlation coefficients. Black lines indicate significant clusters with p < .01. The decoding performance did not consistently surpass the predefined threshold. Pre = Pre-Task, Post = Post-Task, EO = Eyes-Open, EC = Eyes-Closed.


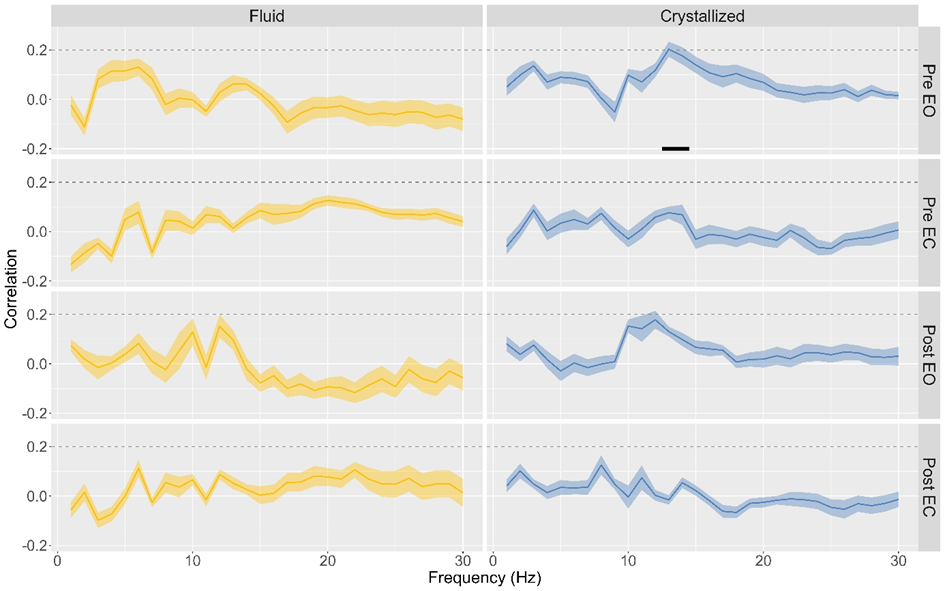


**Figure S4.** Mean decoding performance for fluid and crystallized intelligence, and each condition in the total signal for the female subsample. Shaded areas indicate 95% confidence interval around the correlation coefficients. Black lines indicate significant clusters with p < .01. The decoding performance did not surpass the predefined threshold. Pre = Pre-Task, Post = Post-Task, EO = Eyes-Open, EC = Eyes-Closed.

*
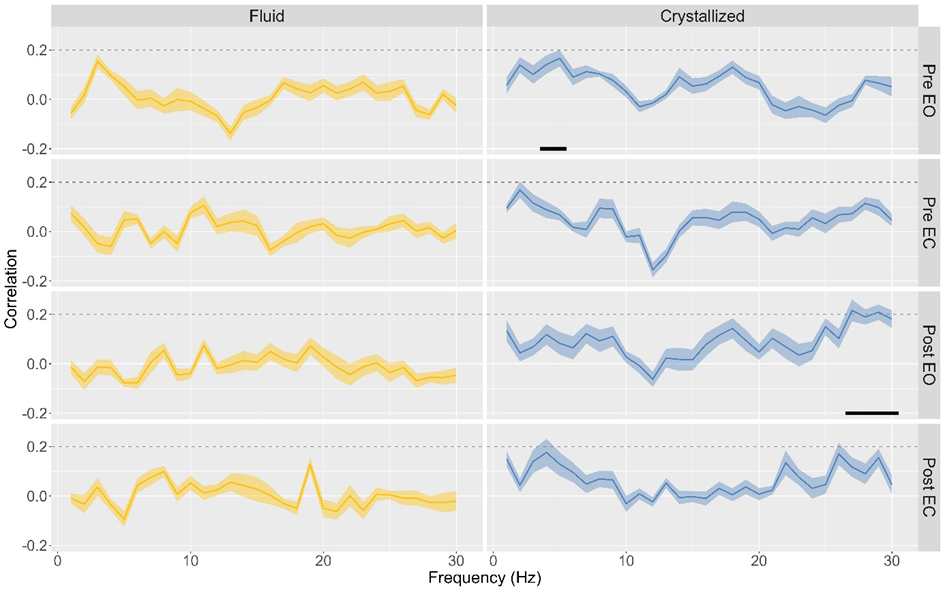
*

**Figure S5.** Mean decoding performance for fluid and crystallized intelligence, and each condition in the periodic signal for the male subsample. Shaded areas indicate 95% confidence interval around the correlation coefficients. Black lines indicate significant clusters with p < .01. The decoding performance did not surpass the predefined threshold. Pre = Pre-Task, Post = Post-Task, EO = Eyes-Open, EC = Eyes-Closed.


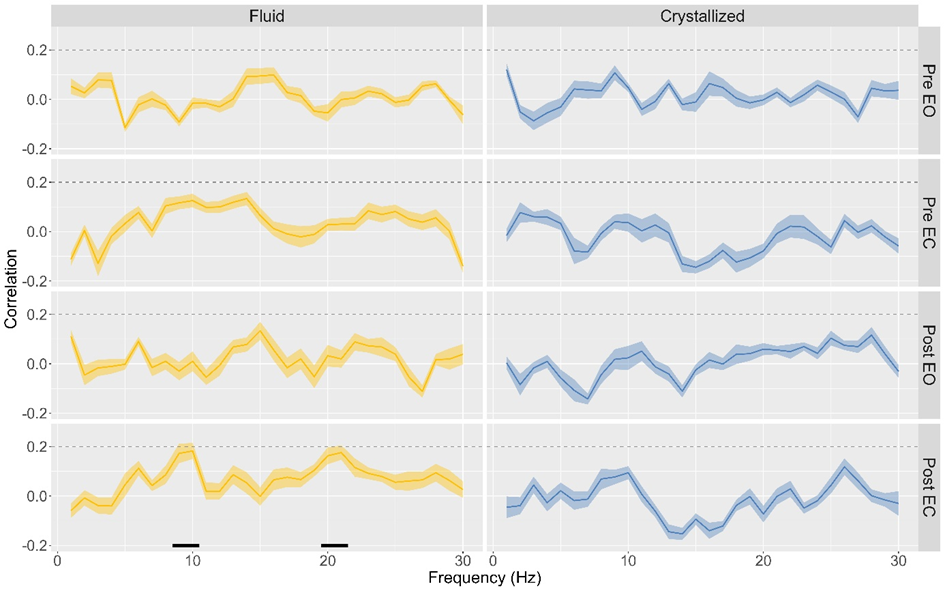


**Figure S6.** Mean decoding performance for fluid and crystallized intelligence, and each condition in the periodic signal for the female subsample. Shaded areas indicate 95% confidence interval around the correlation coefficients. Black lines indicate significant clusters with p < .01. The decoding performance did not surpass the predefined threshold. Pre = Pre-Task, Post = Post-Task, EO = Eyes-Open, EC = Eyes-Closed.
